# Higher expression of cell division cycle-associated protein 5 predicts poorer survival outcomes in hepatocellular carcinoma

**DOI:** 10.18632/aging.103501

**Published:** 2020-07-21

**Authors:** Shengzhong Hou, Xing Chen, Mao Li, Xing Huang, Haotian Liao, Bole Tian

**Affiliations:** 1 Department of Pancreatic Surgery, West China Hospital, Sichuan University, Chengdu, Sichuan, China; 2 Department of Liver Surgery and Liver Transplantation, State Key Laboratory of Biotherapy and Cancer Center, West China Hospital, Sichuan University and Collaborative Innovation Center of Biotherapy, Chengdu, China

**Keywords:** cell division cycle-associated protein 5, prognosis, hepatocellular carcinoma, bioinformatics

## Abstract

The upregulation of cell division cycle associated protein 5 (CDCA5) has been observed in various cancer types. However, the prognostic value of CDCA5 and its underlying mechanism contributing to tumorigenesis in hepatocellular carcinoma (HCC) remain poorly understood. We used tissue microarray (TMA) to evaluate the prognosis of 304 HCC samples based on their CDCA5 expression, and analyzed the genomic features correlated with CDCA5 by using dataset from The Cancer Genome Atlas (TCGA). Compared with adjacent normal tissues, increased expression of CDCA5 was found in HCC tissues. Moreover, higher expression of CDCA5 was associated with inferior OS and DFS outcomes in HCC patients. The enrichment plots showed that the gene signatures in cell cycle, DNA replication and p53 pathways were enriched in patients with higher CDCA5 expression. Meanwhile, statistically higher mutations burdens in TP53 could also be observed in CDCA5-high patients. Integrative analysis based on miRNAseq and methylation data demonstrated a potential association between CDCA5 expression and epigenetic changes. In conclusion, our study provided the evidence of CDCA5 as an oncogenic promoter in HCC and the potential function of CDCA5 in affecting tumor microenvironment.

## INTRODUCTION

Hepatocellular carcinoma (HCC), the most common form of liver cancer, has been ranked among the most common cancers globally [1]. It has been widely known that several risk factors contribute to HCC carcinogenesis, including chronic hepatitis B virus (HBV)/hepatitis C virus (HCV) infection, alcohol abuse, autoimmune hepatitis, diabetes mellitus, obesity, and several metabolic diseases [2]. Despite great advances in the diagnosis and treatment of HCC has been achieved, the prognosis of HCC patients still remains poor over the past decades [3]. Therefore, the main goal of current oncological studies on HCC is understanding the pathophysiological mechanism contributing to the progression of HCC.

Cell division cycle associated protein 5 (CDCA5), which was also known as sororin coded by CDCA5 gene, was initially identified as a substrate of anaphase-promoting complex regulating sister chromatid cohesion [4, 5]. Previous study shows that phosphorylation of CDCA5 at Ser209 by extracellular signal-regulated kinase (ERK) can inhibit the proliferation of lung cancer cells, which is inversed after the induction of exogenous expression of CDCA5 [6]. It has been proved that CDCA5 was significantly up-regulated in various human tumor tissues, including lung cancer, oral squamous cell carcinoma, urothelial cancer and gastric cancer [6–10]. These findings indicate the potency of CDCA5 as a significant oncogenic promoter for cancers. However, the underlying mechanism in which CDCA5 regulate HCC tumorigenesis are still poorly understood.

In our work, we used tissue microarrays (TMA) to evaluate the histopathological features of CDCA5 in HCC tumor samples and analyze the survival outcomes of 304 HCC tumor samples based on CDCA5 expression. Moreover, distinctive genomic features correlated with the expression of CDCA5 were also analyzed by using dataset from The Cancer Genome Atlas (TCGA). The aim of this study is to provide with comprehensive analysis on CDCA5 as a potential target of treatment for HCC, which would address the research gaps in previous studies.

## RESULTS

**Figure 1:**
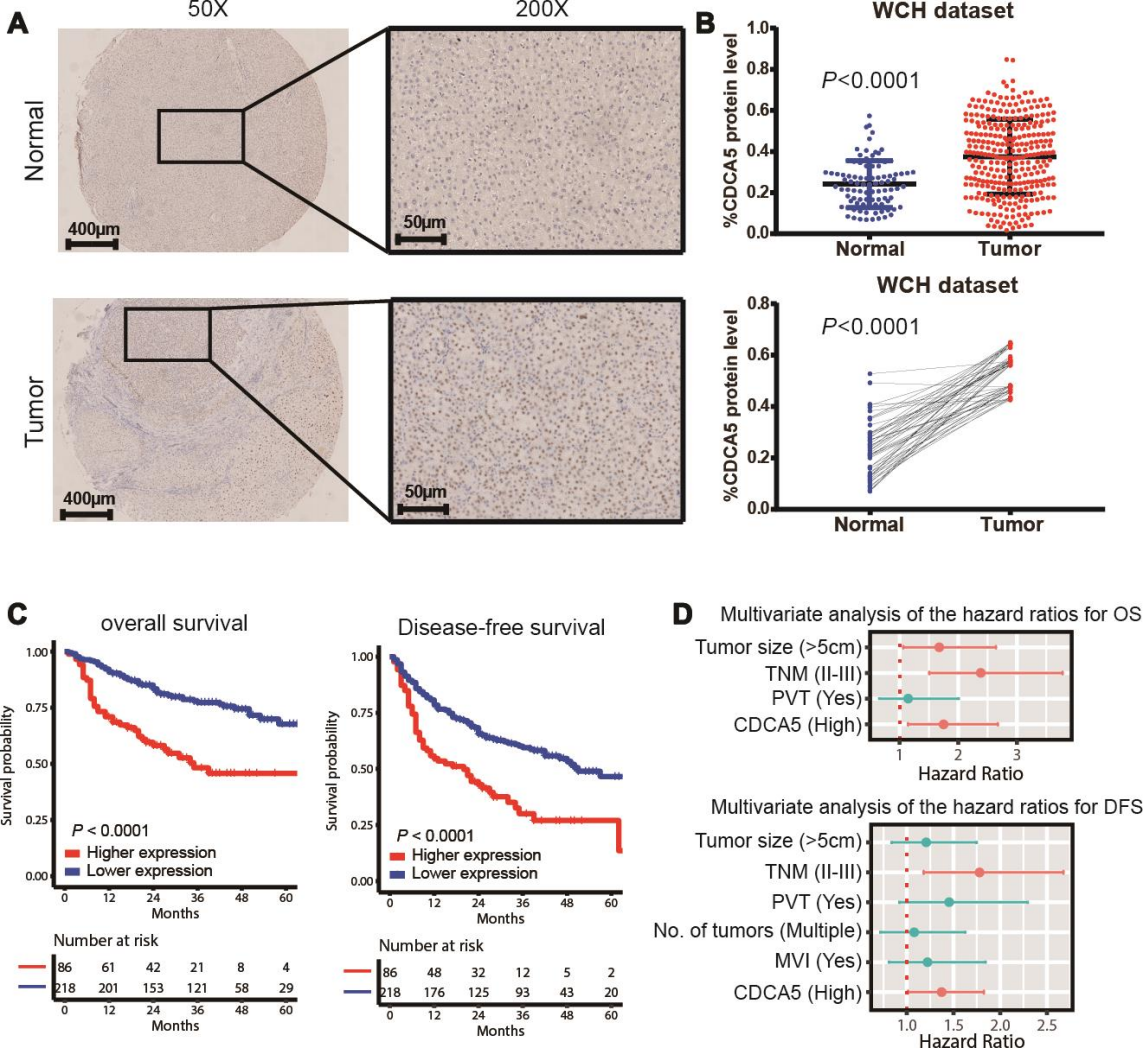
**CDCA5 is upregulated in HCC tissues and predicts poorer survival outcomes.** (**A**) Representative IHC staining of CDCA5 in HCC and paired normal tissues. (**B**) The relative protein level of CDCA5 is significantly higher in HCC tissues than in adjacent normal tissue (upper panel). Data represent the mean±SD. ***, *p*< 0.001. This finding was further validated by comparing CDCA5 expression in tumor and patient-matched adjacent normal tissues (lower panel). (**C**) Higher expression of CDCA5 predicts poorer survival outcomes in patients with HCC. (**D**) Multivariable Cox regression analysis shows that CDCA5 is an independent risk factor for both OS (upper panel) and DFS (lower panel). Independent prognostic factors, including CDCA5 expression and other clinical parameters, were assessed using the multivariate Cox proportional hazards model among the variables found to be significant using univariate analysis. The HRs are presented as the means with 95% confidence interval. Differences with *p*< 0.05 (Red) were considered significant.

In order to evaluate the clinical significance of CDCA5 in HCC, we detected the expression of CDCA5 by using TMA, in which HCC samples (n=304) and matched adjacent normal tissues (n=50) were enrolled. It showed that HCC samples exhibited higher CDCA5 expression than adjacent normal tissues (Figure 1A), which was proved by unpaired and paired *t*-test (Figure 1B). Meanwhile, further validation by GEO datasets also demonstrated the higher CDCA5 expression in tumor tissues (Supplementary Figure 1). By stratifying patients into subgroups based on the best cut-off point set by X-tile software [11], we found that higher CDCA5 expression correlated with both poorer OS and DFS outcomes (Figure 1C). Moreover, a multivariate Cox regression analysis demonstrated the significance of CDCA5 as an independent risk factor for OS and DFS (Figure 1D).

**Figure 2:**
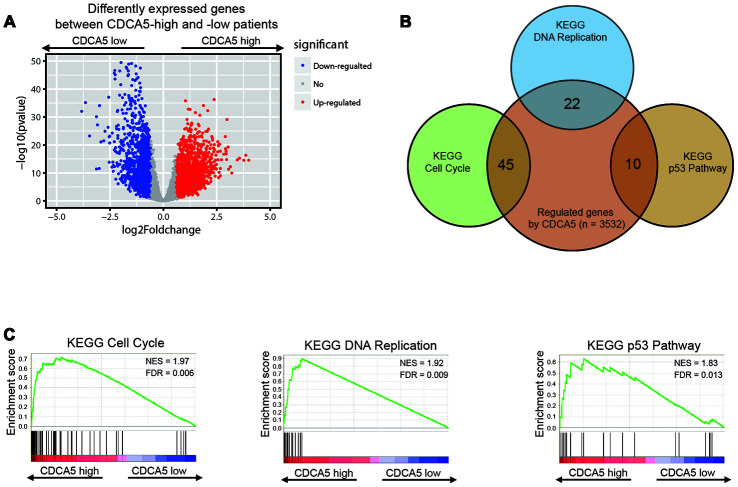
**Identifying differentially expressed genes between CDCA5-high and -low patients.** (**A**) Volcano plot of differential gene profiles between CDCA5-high and -low groups. (**B**) KEGG pathway analysis by GSEA shows that genes involved in cell proliferation, DNA replication and p53 pathway are enriched in CDCA5-high patients. Venn plot demonstrates the overlapping between differentially expressed genes and genes participating in different biological processes. Each circle in the Venn plot represents one set and the number in the overlaid area represents the common genes between the sets. (**C**) GSEA enrichment plots demonstrated gene enrichment results from Figure 3B.

To clarify the potential mechanism of CDCA5 in promoting HCC formation, we used RNAseq to analyze the gene expression in both CDCA5-high and -low groups. To sum up, 1652 genes were up-regulated (≥1.5-fold) and 1885 genes were down-regulated (≥1.5-fold) in CDCA5-high group (CDCA5-low group as reference, Figure 2A). Then, we performed GSEA analysis to find potential pathways in which CDCA5 was involved to affect HCC carcinogenesis. The enrichment plots of KEGG pathways showed that the genes involved in cell cycle, DNA replication and p53 pathway were significantly enriched in patients with higher CDCA5 expression (Figure 2B, 2C). A distinct expression distribution of genes in these 3 pathways showed that CDCA5-high tumors were statistically enriched for these genes (Supplementary Figure 2). These results highly indicate the underlying mechanism in which CDCA5 functions as a down-stream target of p53 pathway and promote HCC carcinogenesis by activating tumor cell proliferation.

**Figure 3:**
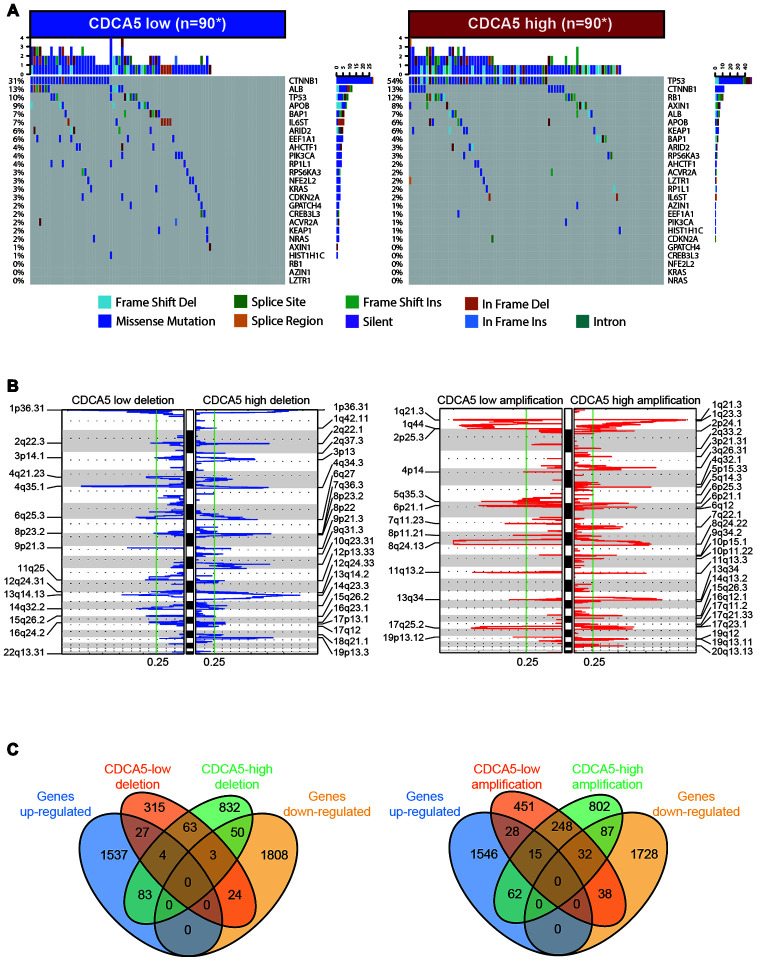
**Association between CDCA5 and mutational signatures, copy number variation in HCC.** (**A**) Significantly mutated genes in HCC subsets stratified by CDCA5 expression. (**B**) GISTIC2.0 analysis identified recurrent somatic copy number alterations in different HCC subsets stratified by CDCA5 expression. (**C**) Venn diagrams demonstrating the number of genes within genomic regions showing significant amplification or deletion, as well as the overlay with significant genes identified from RNAseq in CDCA5-high and -low patients. Each circle in the Venn diagram represents one set and the number in the overlaid area represents the common genes between the sets.

The mutation landscape of driver genes in HCC has been provided by genetic profiling studies based on WGS data from TCGA dataset [12]. To find significant mutation events correlated with CDCA5 expression, we analyzed the mutation profiles characterized for HCC with different CDCA5 expression (high vs. low). It was noticeable that over half of the samples in CDCA5-high group had mutation events in TP53 (Figure 3A), the most renowned tumor suppressor gene proved to suppress tumor development by multiple pathways [13]. Moreover, tumors with higher CDCA5 expression showed statistically higher mutation burdens in TP53 (Table 1). At the same time, higher mutation burdens in tumor suppressor gene RB1 [14] were also observed in CDCA5-high tumors (Figure 3A, Table 1). Intriguingly, higher mutation burdens in CTNNB1 were observed in patients with lower CDCA5 expression (Figure 3A, Table 1). Owing to the fact that the alteration in CTNNB1 can result in the inhibited degradation of the encoded protein (β-catenin) and constitutive activation of β-catenin in HCC [15–17], this finding suggested that there was no association between the activation of Wnt signaling pathway and CDCA5 up-regulation.

**Table 1:** The mutation frequency in CDCA5-low and -high patients.

**Gene**	**CDCA5 low (n=90)**	**CDCA5 high (n=90)**	P* value
**TP53**	9	49	<0.0001
**RB1**	0	11	0.00186
**AHCTF1**	4	2	0.678
**GPATCH4**	2	0	0.477
**CTNNB1**	28	12	0.004124
**AXIN1**	1	7	0.07054
**AZIN1**	0	1	1
**CREB3L3**	2	0	0.477
**ARID2**	5	3	0.7176
**ACVR2A**	2	2	1
**EEF1A1**	5	1	0.2129
**PIK3CA**	4	1	0.3643
**RPS6KA3**	3	3	1
**LZTR1**	0	2	0.477
**ALB**	12	6	0.136
**APOB**	8	5	0.3877
**HIST1H1C**	1	1	1
**NFE2L2**	3	0	0.2442
**KEAP1**	2	5	0.4407
**KRAS**	3	0	0.2442
**BAP1**	6	4	0.7449
**NRAS**	2	0	0.477
**CDKN2A**	3	1	0.6131
**RP1L1**	4	2	0.678
**IL6ST**	6	2	0.2779
**ARID1A**	4	10	0.1641

*, Pearson χ² test *P* value

HCC is characterized by increased genomic instability with extensive copy number alterations [18, 19]. To identify the correlation between CDCA5 expression and CNV, we used GISTIC 2.0 to analyze the copy number amplifications and deletions in various chromosome regions. It showed that a large sum of loci were either significantly amplified or deleted regardless of the expression of CDCA5 (Figure 3B). 1035 genes exhibited copy number deletion in CDCA5-high patients, while the number for CDCA5-low patients was 436 (Figure 3C, left panel). After overlaid with the significantly differentially expressed genes identified by RNAseq, 133 genes within the deletion regions in CDCA5-high patients showed the concordant expression pattern in RNAseq, implying that the differential expression of these genes might be partially owing to copy number deletions. Meanwhile, the number of genes within the aberrantly amplified regions in CDCA5-high patients was 1245 (Figure 3C, right panel), among which 149 genes were also identified as statistically dysregulated according to RNAseq, suggesting that differential expression of these genes may be partially due to the copy number amplifications. In spite of the fact that there were genes exhibiting concordance between RNAseq and CNV results, the majority of the aberrantly expressed genes identified from RNAseq in CDCA5-high patients were not affected by CNV, indicating the independence of differential gene expressions from CNV in patients with higher CDCA5 expression.

**Figure 4:**
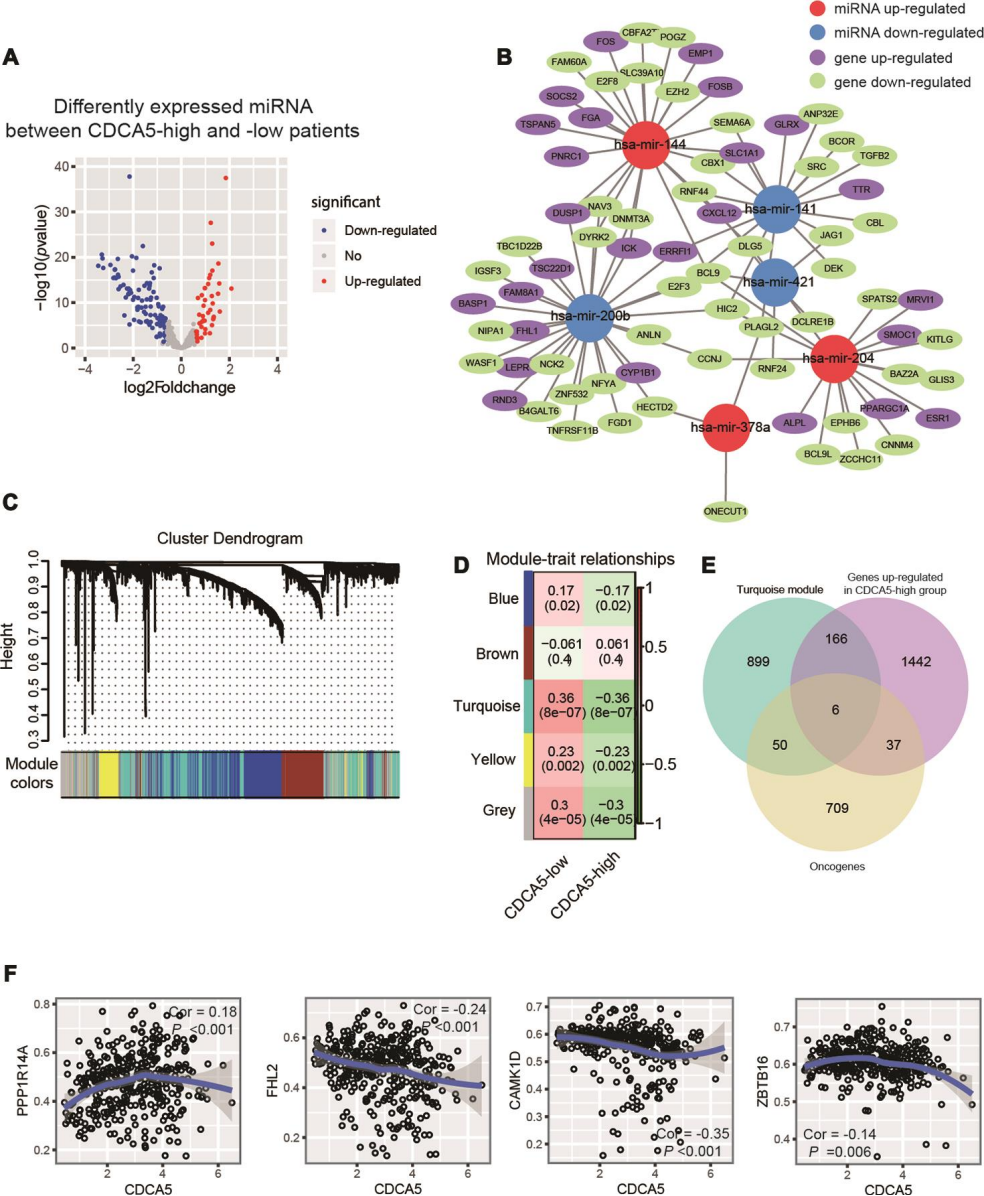
**Integration of epigenetic change and gene expression between CDCA5-high and -low patients.** (**A**) Volcano plot of differentially expressed miRNAs between CDCA5-high and -low groups. (**B**) Regulation of gene expression by miRNA plot as network in cytoscape. (**C**) Dendrogram indicating expression of different gene modules in patients involved in WGCNA analysis. (**D**) Correlation between module eigengenes and the expression level of the CDCA5 (low vs. high). (**E**) Venn diagrams demonstrating the number of genes within module turquoise, as well as the overlay with up-regulated genes identified from RNAseq and oncogenes. (**F**) Local regression curves (Spearman rank correlation) between expression of CDCA5 and 4 oncogenes identified in module turquoise.

MicroRNAs (abbreviated miRNAs) area class of short non-coding RNAs (about 22 nt) which can target mRNAs for cleavage and post-transcriptionally control gene expression [20]. It has been reported that aberrant regulation of miRNA plays a key role in HCC carcinogenesis [21]. In this study, we evaluated the genes potentially regulated by miRNA after the upregulation of CDCA5. A total of 44 up-regulated miRNAs (≥1.5-fold) and 113 down-regulated miRNAs (≥1.5-fold) were detected in CDCA5 group (CDCA5-low group as reference, Figure 4A). By using TargetScan, we identified a total of 97 pairs of miRNA-mRNA interaction, among which 65 pairs had significantly down-regulated genes in CDCA5-high patients (Figure 4B). Notably, hsa-mir-200b negatively regulated the expression of 29 genes. Moreover, 19 genes exhibited negative regulation from more than one miRNA. The interaction network showed that a set of oncogenes, including DNMT3A, TGFB2, CXCL12 and BCL9 [22–25], were potentially regulated by miRNA expressions.

DNA methylation is thought to be an important epigenetic modification regulating gene expression. Previous studies have demonstrated that methylated CpG island of gene promoters will suppress gene expression [26]. It has been reported that dysregulation of DNA methylation significantly correlated with HCC progression [27–29]. To evaluate DNA methylation patterns between CDCA5-low and -high patients, we used WGCNA to cluster methylated genes into different co-methylation modules. The network and the identified modules were illustrated in Figure 4C. Each module was assigned with a unique color identifier, with the remaining poorly connected genes colored gray. Notably, the most significant correlation was observed between CDCA5-high status module turquoise (absolute Pearson correlation coefficient = 0.36 and Bonferroni threshold of *P*= 8e-07*,* Figure 4D). We overlaid oncogenes within these this module with up-regulated genes identified in CDCA5-high patients, so as to find potentially demethylated genes after the up-regulation of CDCA5. The results showed that methylation status of 6 oncogenes were potentially affected by CDCA5 up-regulation (Figure 4E), including TBX3, PPP1R14A, FHL2, CAMK1D, ZBTB16 and AKTIP [30]. Among these 6 genes, the beta values of FHL2, CAMK1D and ZBTB16 showed significant negative correlation with CDCA5 expression, while the beta value of PPP1R14A showed positive correlation with CDCA5 expression (Figure 4F).

## DISCUSSION

Acting as a regulator of sister chromatid cohesion in cell-cycle, CDCA5 exhibit the pro-tumor ability by regulating proliferation process of tumor cells. Consistent with previous studies on various tumor types, higher expression of CDCA5 was found in HCC tumor cells than in adjacent normal tissues. Moreover, higher CDCA5 expression correlated with poorer survival outcomes in HCC patients. By analyzing the differentially expressed genes between CDCA5-low and -high HCC tumor samples, we found that genes involved in cell cycle were significantly enriched in CDCA5-high tumors. This finding indicates that CDCA5 participates in regulating HCC cell proliferation.

The transcription factor p53 plays an essential role in regulating cell cycle and is the most important tumor suppressor widely known [31]. When exposed to cellular stress signaling including DNA damage and oncogenic pressure, p53 can be activated by phosphorylation of its protein and posttranslational modifications, which result in the up-regulation of p53 target genes involved in DNA repair, apoptosis and cell-cycle arrest. Inactivation of p53 through either mutation or alterations in related pathways has been regarded as a hallmark of every tumor types [32]. According to our GSEA analysis, several p53 downstream genes were significantly up-regulated in CDCA5-high group, including genes involved in cell-cycle (CDK4, CDKN2A, CDK2, CCNB2, CDK1 and CCNB1), apoptosis (BAX) and DNA synthesis (RRM2). Considering that CDCA5-high patients had statistically higher mutation burdens in TP53 (Figure 4A, Table 1), this result indicated a potential loss of function correlated with TP53 mutation contributing to the dysregulation of genes involved in p53 pathway, thus promoting the expression of CDCA5 and HCC tumorigenesis.

As the first identified tumor suppressor gene, the retinoblastoma gene RB1 has been proved to regulate the various biologic processes, including cell cycle progression, terminal differentiation and DNA replication [33]. RB1 mutation can cause the inactivation of the gene product pRB by exempting normal cells to exit cell cycle, which leads to high susceptibility of normal cells to oncogenic proliferation. This can be observed in almost all familial and sporadic forms of retinoblastoma and other human cancers at variable frequencies [14]. In our work, we also showed that the proportion of patients with RB1 mutations in CDCA5-high group was statistically higher than that in CDCA5-low group, suggesting that inactivation of pRB can lead to abnormally up-regulation of CDCA5 during carcinogenesis, which contribute to HCC tumor cell proliferation.

At this moment, it is evident that miRNA is a key regulator in carcinogenesis. During the process of tumor formation, mature miRNA is generated from two-step cleavage of primary miRNA (pri-miRNA), which incorporates into a large protein complex called RNA-induced silencing complex (RISC) (48-50). By identifying significant miRNAs potentially regulated by CDCA5 expression, we found that hsa-mir-144, a tumor suppressor miRNA in various cancer types including HCC [34–36], was significantly up-regulated in CDCA5-high patients. Meanwhile, another tumor suppressor miRNA, hsa-mir-200b, [37–39] was down-regulated in CDCA5-high patients. Moreover, multiple synchronizations existed between the expressions of miRNAs and their target genes. These findings indicated the existence of a highly complicated regulatory network by miRNA expressions along with the upregulation of CDCA5.

While the direct impact of DNA methylation on tumor suppressor genes has been well established for decades [40], a prevailing alternative hypothesis has aroused great interest in hypomethylation as a significant epigenetic alteration resulting in the transcriptional activation of oncogenes [41]. Although there are genes identified to be activated due to promoter hypomethylation in cancers, their oncogenic roles still remain poorly understood [42]. Since the methylation beta value of 3 oncogenes up-regulated in CDCA5-high patients, including FHL2, CAMK1D and ZBTB16, showed significantly negative associations with CDCA5 expression, our study indicated the transcriptional activation of these genes by DNA demethylation, which was potentially induced by CDCA5 up-regulation. Meanwhile, the positive correlation between the beta value of oncogene PPP1R14A and CDCA5 expression also suggested the competition between these 2 genes in gene expression. Further research will be inspired to detect the function of CDCA5 as a DNA methylation regulator.

In conclusion, our study provided the evidence of CDCA5 as an oncogenic promoter in HCC and its potential function in affecting tumor microenvironment. The results in this work revealed the underlying mechanism in which CDCA5 up-regulation contributed to the poorer survival outcomes in HCC patients. Moreover, our study highlighted the potential value of CDCA5 targeted therapy in future clinical practice.

## MATERIALS AND METHODS

### Patients and samples

A total of 304 HCC patients undergoing hepatectomy between 2007 and 2012 in West China Hospital were included in this study. Tissue microarrays were constructed as previously described [43]. Tumor staging classification was carried out according to the 7^th^ AJCC TNM Staging for Liver and Intrahepatic Bile Duct Malignancies. The characteristics of tumor samples, including differentiation, size, number of nodules, vascular invasion and Ishak fibrosis score of the adjacent liver tissue were evaluated by two pathologists specializing in hepatic diseases. The primary end point of this study was overall survival (OS), which was defined as the time from the date of surgery to the date of death without regard to the cause of death. The secondary end point was disease-free survival (DFS) defined as the time from the date of surgery to the time of the first event (recurrence, progression, death).

This study, including any relevant details, was approved by the ethics committee of West China Hospital. All the patients included in this study were over 18 years old and informed consent was obtained from study participants according to the regulations of the committee. Patients' names and other HIPAA identifiers have been excluded from this study. We confirm that all experiments were performed in accordance with relevant guidelines and regulations.

### Evaluation of CDCA5 staining

The tissue core punched from a representative tissue area of the formalin-fixed, paraffin-embedded (FFPE) slide of each HCC sample was selected to construct the TMAs. H&E staining on TMAs were performed as previously described [44]. Immunohistochemical (IHC) staining was performed as previously described [43] by using a specific anti-CDCA5 antibody (1/500, Abcam). Images of CDCA5 staining were viewed and captured using the NDP.view.2 software program. Slides were reviewed by two experienced pathologists who were blind to the clinical parameters. We evaluated the positive staining of CDCA5 in tumor cell nuclei from 5 respective areas in each TMA dot at 20× magnification and recorded the percentage of positively stained cells in each area. The mean value from the 5 areas was used for further analyses.

### RNAseq gene expression analysis

Raw counts of gene expression from RNAseq were downloaded from the TCGA data portal (https://portal.gdc.cancer.gov/) for the differential gene expression analysis. Total raw read counts per gene were divided by the gene’s maximum transcript length to represent a coverage depth estimate, which were then scaled to a total depth of 106 per sample and can be interpreted as transcripts per million (TPM) [45]. Statistical ranking for CDCA5 expression by the top and bottom quartiles were defined as CDCA5-high and CDCA5-low, respectively. Differential gene expression analysis between CDCA5-high and CDCA5-lowpatients across TCGA datasets was calculated using the R package edgeR, which determines the differential gene expression by accounting for variability through an over-dispersed Poisson model and moderating the degree of over-dispersion by Empirical Bayes methods [46]. Genes with counts per million (CPM) larger than 1 across at least 91 samples (half of all samples) were included for differential gene expression analysis. Genes with the adjusted *p* value less than 0.05 and the absolute FC larger than 1.5 were considered to be statistically significant. KEGG pathway analysis on the aberrantly expressed genes between CDCA5-high and CDCA5-lowpatients was performed based on gene set enrichment analysis (GSEA) as previously described [47]. Terms with a false discovery rate (FDR) < 0.05 were considered statistically significant. Normalized gene expression data and the corresponding clinical data were also obtained from TCGA data portal.

### Mutation and copy number variation analysis

Significantly mutated genes (SMGs) were defined by running the Mutational Significance in Cancer (MuSiC Genome Suite) in different subtypes of HCC (CDCA5-high vs. CDCA5-low). MuSiC identifies genes with significantly higher mutation rates than the background mutation rate (BMR) to find SMGs across the entire sample population. The threshold for significance was a FDR of 0.1. Mutational spectra across the entire study population from the TCGA dataset were determined as previously described [12]. Copy number variation (CNV) data was downloaded from GDAC Firehose and separated into different datasets according to the expressions of CDCA5. Investigation into significant amplification or deletion events in the regions of the genome was conducted through the use of GISTIC 2.0, a revised computational program to identify somatic copy number alteration by investigating the frequency and amplitude of observed events [48]. Meanwhile, genes within the significant genomic regions were further analyzed to determine the overlay with those differentially expressed and identified from RNAseq.

### Integration of gene expression and epigenetic change

To investigate the potential genes regulation by miRNA, we focused on aberrantly expressed miRNA (adjusted *p* value < 0.05, absolute FC > 1.5) and the significant differential gene selected from RNAseq between CDCA5-high and CDCA5-low patients. Since miRNAseq only provided the expression level of the stem loop, the stem loop’s expression level was considered as the mature miRNA. The correlation between miRNA and the regulated genes was analyzed by TargetScan [49–53].

Preprocessed methylation data (mean beta values, level 3) were downloaded from Broad Firehose (http://gdac.broadinstitute.org/). Weighted Gene Co-Expression Network Analysis (WGCNA) [54] was conducted to identify groups of methylated genes (modules) involved in patients with different CDCA5 status (high vs. low) as previously described [55]. Genes in the modules showing statistically positive correlation with CDCA5-high status were further analyzed to determine the overlay with those down-regulated in CDCA5-high group and oncogenes identified by ONGene [30].

### Gene expression omnibus (GEO) data

Microarray gene expression data of HCC samples were downloaded from the GEO database (accession numbers GSE1898, GSE54236, GSE64041) [56–59]. The R package “GEOquery” was used to extract the expression values of genes.

### Statistical analysis

Statistical analyses and graphics were undertaken using R version 3.5.1. Student's *t*-test and Pearson χ² test were used for the univariate analyses where appropriate. Survival rates of expression level (high vs. low) were estimated by the Kaplan-Meier method with Rothman CIs. Survival curves were compared with the log-rank test. The hazard ratio (HR) and 95% CI associated with the expressions of CDCA5 were estimated through a multivariable Cox regression model adjusted for TNM stage (I vs. II-III), portal vein thrombus (no vs. yes), number of tumors (single vs. multiple), tumor size (≤5cm vs. >5cm) and microvascular invasion (no vs. yes). A *p* value < 0.05 was considered statistically significant.

## Supplementary Material

Supplementary Figures
