# Supplementary material for: Higher expression of cell division cycle-associated protein 5 predicts poorer survival outcomes in hepatocellular carcinoma
Source: Aging (Albany NY). 2020 Jul 21;12(14):14542–55. doi: 10.18632/aging.103501 (PMC7425481; doi:10.18632/aging.103501)
Supplement: Supplementary Figures [file aging-12-103501-s001..pdf]

SUPPLEMENTARY FIGURES

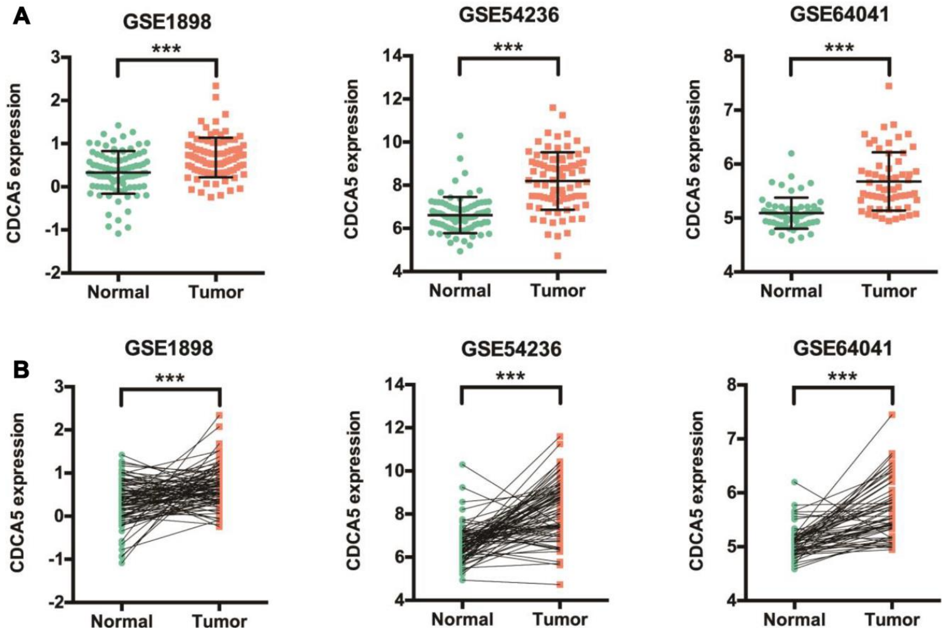

Supplementary Figure 1. The relative mRNA expression of CDCA5 in GEO datasets. (**A**) The relative mRNA level of CDCA5 is significantly higher in HCC tissues than in adjacent normal tissue. Data represent the mean ± SD. ***, $P < 0.001$. (**B**) The relative mRNA level of CDCA5 is higher in HCC than patient-matched adjacent normal tissues. ***, $P < 0.001$.

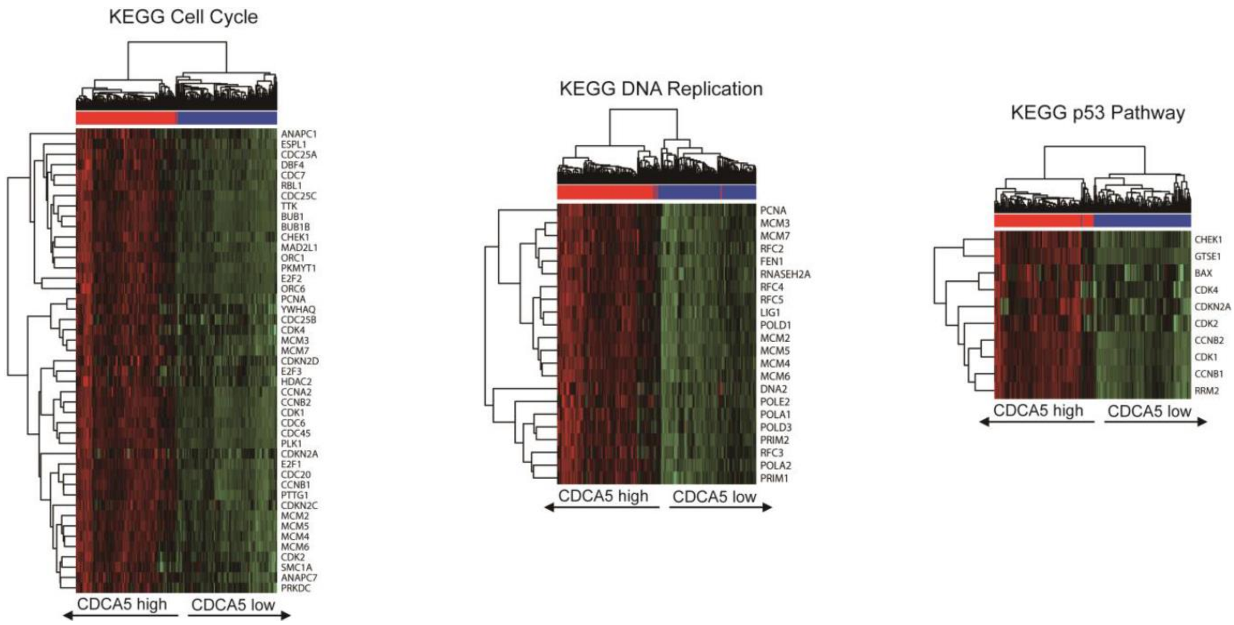

Supplementary Figure 2. Enrichment clustering of genes involved in cell cycle, DNA replication and p53 pathway between CDCA5-high and -low patients.
